# Cognitive Dysfunction in Fibromyalgia: Prevalence and Independent Predictors—A Case–Control Study Using the Montreal Cognitive Assessment Scale

**DOI:** 10.3390/brainsci16010068

**Published:** 2026-01-01

**Authors:** Sofia Ferreira Azevedo, Inês Genrinho, Joana Saldanha, Inês Cunha

**Affiliations:** 1 Rheumatology Department, Unidade Local de Saúde da Região de Aveiro, Av. Artur Ravara, 3800-164 Aveiro, Portugal; 11337@chbv.min-saude.pt; 2 Aveiro Rheumatology Research Centre, 3800-164 Aveiro, Portugal; 3 Rheumatology Unit, Unidade Local de Saúde Tondela-Viseu, 3460-541 Viseu, Portugal; igenrinho@gmail.com; 4 Physical and Rehabilitation Medicine Department, Unidade Local de Saúde da Região de Aveiro, 3800-164 Aveiro, Portugal; 72159@chbv.min-saude.pt

**Keywords:** cognitive dysfunction, fibromyalgia, Montreal Cognitive Assessment Scale

## Abstract

**Background:** Cognitive dysfunction is a frequent but under-recognized feature of fibromyalgia (FM). Its prevalence varies widely across studies, and independent clinical predictors remain uncertain. This study aimed to determine the prevalence of cognitive dysfunction in FM patients compared with healthy controls and identify independent associated factors. **Methods:** We conducted a case–control study including 47 adult female patients with FM (2016 ACR criteria) and 19 age- and sex-matched healthy controls. Sociodemographic and clinical data were collected. Cognitive function was evaluated using the Montreal Cognitive Assessment (MoCA), with cognitive dysfunction defined as MoCA < 26. Pain (VAS), fatigue (VAS and FACIT-F), anxiety and depression (HADS), sleep quality (PSQI), and disease impact (FIQ-P) were assessed. Univariate analysis was followed by binary logistic regression to identify independent predictors of cognitive dysfunction and multiple linear regression to explore associations with MoCA score. **Results:** Cognitive dysfunction was present in 72.3% of FM patients versus 5.3% of controls (*p* < 0.001). FM patients had significantly worse pain scores, fatigue levels, psychological distress, sleep quality, and quality of life (all *p* < 0.001). In FM patients, MoCA scores correlated inversely with pain (r = −0.34), anxiety (r = −0.34), depression (r = −0.48), disease impact (r = −0.43), and sleep disturbance (r = −0.48), and positively with FACIT-F (r = 0.37) and EQ-5D-5L (ρ = 0.60). In multivariate analysis, higher FIQ-P scores were independently associated with cognitive dysfunction [adjusted OR1.18; 95% CI (1.06–1.30); *p* < 0.01]. Pain severity [adjusted B = −0.40; 95%CI (−0.64–0.15; *p* < 0.01)] and depression [adjusted B = −2.60; 95% CI (−4.12–1.04; *p* = 0.001)] were independently associated with lower MoCA scores. **Conclusions:** Cognitive dysfunction is highly prevalent in FM and is independently associated with pain severity, depressive symptoms, and disease impact.

## 1. Introduction

Fibromyalgia (FM) is one of the leading causes of chronic widespread pain and the third most common musculoskeletal condition, after low back pain and osteoarthritis [1]. Its global prevalence is estimated at approximately 2.7%, with a female-to-male ratio of about 3:1. In Portugal, data from the EpiReumaPt study report a prevalence of 1.7% in the general population, with a striking gender difference (3.1% in women vs. 0.1% in men) [2].

The clinical conceptualization of FM has evolved since the 19th century. Smythe and Moldofsky were the first to coin the term “fibromyalgia” after describing tender points as a hallmark of the condition [3]. In 1990, the American College of Rheumatology (ACR) published the first classification criteria, which were subsequently updated in 2010/2011 and revised in 2016 [4,5,6]. The 2016 criteria rely on the Widespread Pain Index (WPI), the Symptom Severity Scale (SSS), and the presence of generalized pain for at least three months [6]. These revisions reflect a shift from a tender-point–based concept to a symptom-based framework that better captures the multisystem nature of FM beyond pain (e.g., fatigue, sleep, and cognitive complaints).

The negative impact of FM on health-related quality of life is well established and reflected not only in the high direct costs related to healthcare utilization but also in the considerable indirect societal burden arising from reduced productivity and work disability [7,8].

Although chronic widespread pain remains the hallmark feature of FM, the syndrome is clinically heterogeneous, encompassing fatigue, sleep disturbances, mood disorders, and cognitive dysfunction [9,10,11]. The latter—commonly referred to as “fibro fog”—is characterized by impairments in attention, memory, executive function, and processing speed. Cognitive complaints are highly prevalent, affecting up to 70–80% of patients, and exert a significant impact on daily functioning and overall quality of life [11,12]. Although being recognized as an important manifestation associated with FM, there are no formal recommendations regarding its systematic screening, nor are specific assessment tools currently applied in clinical practice guidelines. Furthermore, the assessment of cognitive dysfunction in FM has been inconsistent across studies, often relying on heterogeneous neuropsychological tests not easily applicable in clinical settings. In routine care, comprehensive neuropsychological batteries are often time-consuming, may require trained personnel and standardized testing conditions, and are therefore difficult to implement systematically, particularly in high-volume outpatient settings [13].

The pathophysiology of cognitive dysfunction in FM is multifactorial. Central sensitization mechanisms and altered pain processing in the brain appear to interact with cognitive networks, while impaired sleep, chronic fatigue, affective symptoms (depression and anxiety), and stress further contribute to the deterioration of cognitive performance [14,15,16]. In addition, some studies suggest that neuroinflammatory processes and altered neurotransmitter systems may also play a role [17].

Recognizing the significant burden imposed by cognitive symptoms in FM, identifying the factors that contribute to their occurrence is of utmost importance. Pinpointing predictors such as depression, anxiety, fatigue, poor sleep quality, or higher pain severity may provide the basis for tailored therapeutic strategies. In line with this rationale, the present study aimed to characterize cognitive dysfunction in FM and to determine its predictors.

## 2. Materials and Methods

### 2.1. Study Design

This was a case–control study conducted between May and June 2022 at the Rheumatology Department of Unidade Local de Saúde da Região de Aveiro (Portugal), including adult patients with FM, followed in our Centre and voluntarily recruited, and a control group of healthy participants matched by age and sex, recruited from the same geographic area. Eligible FM patients attending the Rheumatology outpatient clinic during the study period were invited to participate consecutively during scheduled clinic visits. Controls were screened through a brief questionnaire to exclude a history of FM, chronic widespread pain, inflammatory rheumatic disease, neurological disease, or other conditions likely to affect cognitive performance.

The study was approved by the health committee of our Centre and complied with the tenets of the Declaration of Helsinki for biomedical research. Written informed consent was obtained for each participant.

Inclusion Criteria

– Diagnosis of FM according to the 2016 ACR Criteria;
– Age ≥ 18 years;
– Able to speak and read Portuguese;
– Able to give informed consent.

Exclusion Criteria

– Diagnosis of other diseases impacting cognitive function, including major psychiatric disorders, based on medical records and/or clinical interview;
– Diagnosis of other diseases impacting quality of life, fatigue, pain, or sleep.

### 2.2. Data Collection

Sociodemographic and clinical data were collected. Sociodemographic variables included age, sex, education status, and working status. Gynecologic and obstetric history (gestation and menopause) was recorded when applicable.

Lifestyle behaviors such as alcohol consumption, tobacco abuse, and regular physical exercise (>150 min per week) were assessed.

Clinical information included body mass index (BMI), disease duration from symptom onset and from diagnosis (not applicable to the control group), as well as the presence of possible accompanying manifestations as headaches, memory loss, irritable bowel syndrome, temporomandibular joint dysfunction, restless legs syndrome, and joint hypermobility (Beighton score ≥ 4 or 5 for patients aged ≥ 50 and <50 years, respectively).

Data regarding chronic medication (analgesic/neuromodulators/antidepressants/anxiolytics/muscle relaxants) use were also obtained.

All participants underwent a structured assessment that included the following:

Pain and fatigue intensity: measured by the Visual Analog Scale (VAS), a scale ranging from zero to 10, where zero represented the absence of pain/fatigue, and 10 represented the worst pain/level of fatigue the patient could possibly imagine.

Fatigue intensity: evaluated using the Functional Assessment of Chronic Illness Therapy—Fatigue (FACIT-F). It consists of 13 items, each scored on a five-point Likert scale ranging from zero (“Not at all”) to four (“Very much”), with a total score ranging from zero to 52. Higher scores indicate less fatigue and better functional status, whereas lower scores reflect greater fatigue severity [18].

Anxiety and depression: assessed through the Hospital Anxiety and Depression Scale (HADS). It consists of two subscales (HADS-A: anxiety; HADS-D: depression) with seven items each and scores ranging from zero to three. We applied a cut-off score of ≥eight points on each scale because it showed good sensitivity and specificity for determining the presence of anxiety or depressive symptoms [19].

Cognitive function: assessed with the Montreal Cognitive Assessment (MoCA). It evaluates multiple cognitive domains, including attention, memory, language, visuospatial abilities, executive functions, and orientation, with a maximum score of 30. Cognitive dysfunction was assumed for a MoCA < 26 points. This threshold was selected based on standard MoCA recommendations for screening purposes [20].

Sleep quality: evaluated with the Pittsburgh Sleep Quality Index (PSQI). It consists of 19 items grouped into seven components (subjective sleep quality, latency, duration, efficiency, disturbances, use of sleep medication, and daytime dysfunction), generating a global score ranging from 0 to 21, with higher scores indicating poorer sleep quality. Poor sleep quality was considered for a PSQI > 5 [21].

Disease’s impact: measured through Fibromyalgia Impact Questionnaire validated for the Portuguese population (FIQ-P) (not applicable to the control group). The FIQ consists of 10 subscales assessing physical function, number of days feeling unwell, work absenteeism, social functioning, fatigue, morning tiredness, stiffness, anxiety, and depression. The total score ranges from 0 to 100, with higher scores indicating a greater negative impact on health status [22].

### 2.3. Statistical Analysis

A descriptive analysis was performed using means/medians and standard deviation (SD)/interquartile range (IQR) for continuous data, and frequencies and percentages for qualitative variables. The Shapiro–Wilk test was performed to evaluate the normality of the distributions.

A univariate analysis, with chi-square for categorical variables and parametric t-student and non-parametric Wilcoxon tests for continuous variables, was performed to compare FM patients with the control group. Correlations between MoCA scores and continuous variables were assessed with Pearson or Spearman correlation coefficients, as appropriate.

Subsequently, FM patients were divided into two groups according to the presence or absence of cognitive dysfunction. A univariate analysis, with chi-square for categorical variables and parametric Student’s *t*-test and non-parametric Wilcoxon tests for continuous variables, was performed. Lastly, a multivariate analysis, adjusted for anxiolytic, antidepressant, and neuromodulator medication, was performed, including a logistic regression model, to identify independent predictors of cognitive dysfunction in these patients and a multiple linear regression to identify independent associations between MoCA and different variables with significant association on univariate analysis. Multicollinearity among predictors was assessed using tolerance and variance inflation factors (VIF). Values of VIF > 5 (and/or tolerance < 0.20) were considered indicative of potentially problematic multicollinearity. Significance was set at α = 0.05. Statistical analysis was performed with SPSS^®^ software, version 27.

## 3. Results

A total of 66 women were included in the study, comprising 47 patients with FM and 19 healthy controls. Sociographic and clinical data are presented in Table 1. The mean age of the participants was 47.6 ± 10.7 years, with no significant differences between groups (*p* = 0.18). There were also no significant differences between groups regarding educational attainment, with 74.5% of FM patients and 57.9% of controls having completed high school or university education (*p* = 0.24). Unemployment was more frequent among the FM group (*p* < 0.01). Considering medication use, a significantly higher proportion of FM patients were under chronic analgesic therapy compared with controls, including regular use of NSAIDs (68.1% vs. 10.5%), antidepressants (74.5% vs. 15.8%) and sleep-inducing agents (42.6% vs. 5.3%) (*p* < 0.001).

In both groups, less than 50% of participants were compliant with regular exercise (>150 min/week).

Regarding clinical characteristics, FM patients presented substantially higher mean VAS pain scores compared with controls (7.0 [6.0–8.5] vs. 0.00 [0.00], *p* < 0.001), and significantly greater fatigue intensity as assessed by the VAS and FACIT-F questionnaire [8.0 [1.5] vs. 0.00 [0.00] and 19.00 [11.00] vs. 48.00 [11.00], respectively (*p* < 0.001)]. Health-related quality of life, measured by EQ-5D-5L, was markedly lower in FM patients (0.64 [0.9] vs. 1.00 [0.09], *p* < 0.001). Furthermore, FM patients reported significantly higher levels of psychological distress, with elevated HADS anxiety and depression subscores compared with controls (11.72 ± 4.08 vs. 5.53 ± 3.13, and 10.23 ± 4.94 vs. 3.32 ± 3.76; *p* < 0.001), a higher frequency of relevant depressive and anxiety symptoms (*p* < 0.001).

Poor sleep quality was present in all FM patients and in about 68% of controls (*p* < 0.001).

Cognitive dysfunction was present in 34 FM patients (72.3%) compared with only one control participant (5.3%) (*p* < 0.001).

When exploring the relationship between cognition and other disease parameters in patients with FM, MoCA scores demonstrated weak-to-moderate inverse correlations with VAS pain (r = −0.34, *p* = 0.02), HADS anxiety (r = −0.34, *p* = 0.02), HADS depression (r = −0.48, *p* = 0.01), FIQ-P scores (r = −0.43, *p* < 0.01), and PSQI (r = −0.48, *p* < 0.01) Positive weak-to- moderate correlations were observed with FACIT-F (r = 0.37, *p* = 0.01) and EQ-5D-5L scores (ρ = 0.6, *p* < 0.001). Those with cognitive impairment exhibited higher VAS pain scores (7.8 ± 1.6 vs. 5.9 ± 1.7, *p* = 0.03), greater disease impact as measured by FIQ-P (77.5 ± 10.1 vs. 55.2 ± 11.6, *p* < 0.001), and poorer EQ-5D-5L scores (0.44 ± 0.11 vs. 0.63 ± 0.12, *p* < 0.001), compared with patients without cognitive dysfunction.

In binary logistic regression, cognitive dysfunction remained independently associated with higher FIQ-P scores [adjusted OR1.18; 95% CI (1.06–1.30); *p* < 0.01]. In multivariate linear regression, higher pain severity was independently associated with lower MoCA scores [adjusted B = −0.40; 95%CI (−0.64–0.15; *p* < 0.01)]. No evidence of problematic multicollinearity was observed (maximum VIF = 3.5; minimum tolerance = 0.28). The presence of depression was also independently associated with worse cognitive performance, with patients scoring on average 2.6 points lower on the MoCA compared to non-depressed patients [adjusted B = −2.60; 95% CI (−4.12–1.04; *p* = 0.001)].

## 4. Discussion

Our study demonstrates that cognitive dysfunction is a highly prevalent and clinically relevant feature of FM, with over 70% of FM patients demonstrating poor performance in the MoCA. Our results align with current evidence on cognitive impairment in these patients, often referred to as “fibro fog”, with studies reporting prevalences from 50% to 80%. This term describes the constellation of cognitive symptoms frequently reported by FM patients, including forgetfulness, difficulty concentrating, slowed information processing, and mental fatigue [12,13].

The Montreal Cognitive Assessment (MoCA) was developed in 2005 by Nasreddine et al. as a brief screening tool designed to detect mild cognitive impairment, offering broader coverage of executive function, attention, memory, and visuospatial skills compared to traditional instruments such as the Mini-Mental State Examination (MMSE) [20]. The use of this tool in FM patients was evaluated and supported by previous studies suggesting its superior diagnostic accuracy in FM and other conditions characterized by mild cognitive impairment. In a comparative study by Murillo-García et al. (2021), the MoCA outperformed the MMSE in detecting cognitive deficits in FM, demonstrating a lower ceiling effect (5.6% vs. 25%) and stronger correlations with functional performance, particularly during dual-task mobility tests [23]. Similarly, Elkana et al. (2022) compared MoCA results with computerized cognitive batteries and reported moderate correlations across domains, supporting MoCA’s validity in screening cognitive dysfunction in FM [24]. In this study, we operationalized cognitive dysfunction using a MoCA score < 26, a threshold commonly used for screening purposes [20]. We acknowledge, however, that MoCA performance and the most appropriate cut-off may vary across populations, particularly according to age and educational attainment, and that dichotomizing a continuous cognitive measure may obscure clinically relevant variability. Importantly, our sample was relatively highly educated, with more than 70% of participants having ≥12 years of education, which likely reduces bias related to low educational attainment and supports the plausibility of applying this screening threshold in our context.

To our knowledge, although previous case–control studies have consistently demonstrated poorer cognitive performance in FM patients compared with healthy controls, using a variety of neuropsychological tests and domain-specific batteries, we are the first to employ the MoCA as a primary screening tool in this population [25,26,27,28].

The independent clinical association between pain, depression, and cognitive dysfunction observed in our study, also reported by others [16,27], can be partly explained by the complex physiopathology of FM. Central sensitization plays a pivotal role, with altered nociceptive processing leading to hyperactivation of pain-related brain regions, disrupted connectivity, and reduced recruitment of cortical areas involved in pain anticipation and inhibition [29,30,31]. At a neurochemical level, FM patients present increased cerebrospinal levels of substance P and glutamate—especially within the insula, anterior cingulate cortex (ACC), and prefrontal cortex (PFC)—together with reduced availability of µ-opioid receptors and lower concentrations of serotonin, noradrenaline, and dopamine [32,33,34,35]. These changes contribute to neuronal hyperexcitability, heightened pain sensitivity, and inefficient top-down modulation [31]. Importantly, the same brain networks involved in pain modulation also subserve cognitive control and emotional regulation. Insular hyperactivation in FM prioritizes pain-related processing, limiting resources for attention and working memory, while ACC dysfunction impairs conflict monitoring and cognitive flexibility. Disrupted PFC connectivity with parietal and limbic regions contributes further to slowed processing speed and executive dysfunction [11,15,36]. Elevated glutamate and glial-driven neuroinflammation exacerbate synaptic inefficiency, reinforcing these deficits [37]. Moreover, abnormal PFC–limbic interactions help explain the frequent co-occurrence of depression and anxiety, which further amplify attentional bias toward pain and emotional distress [11]. Together, these converging mechanisms offer a plausible explanation for our findings: fibro fog likely results from overlapping disturbances in pain, cognitive, and emotional networks, rather than as an isolated phenomenon. Nevertheless, other studies reported contrasting results, demonstrating no association between significant depressive symptoms and cognitive impairment in FM patients [15,26]. These disparities may, in part, however, be justified by the different neurocognitive tests applied across studies. Also, although participants with a prior diagnosis of major psychiatric disorders were excluded, a substantial proportion of patients reported clinically relevant depressive symptoms. Therefore, we cannot entirely rule out the possibility that some individuals with undiagnosed psychiatric conditions may have been included in the study, and that influence the results obtained.

From a clinical perspective, the identification of pain severity and depressive symptoms as independent predictors of cognitive dysfunction reinforces the need for a multidisciplinary approach in the management of FM. Beyond analgesic strategies, psychological and psychiatric interventions, as well as cognitive training and rehabilitation, may play a role in mitigating the burden of cognitive dysfunction.

Although univariate analysis in our study suggested an association between cognitive dysfunction and sleep quality, this relationship did not remain significant in the multivariate model. The contribution of sleep disturbances to cognitive impairment and executive dysfunction in FM has been less explored than that of mood disorders, and the available evidence remains inconsistent. For instance, Grace et al. [30] and Fernández-Palacios et al. [26] reported no significant effects of sleep quality on cognitive performance, whereas Miró et al. found that sleep problems predicted alertness-related cognitive impairment [29]. In our cohort, all patients presented PSQI scores consistent with poor sleep quality, which may justify the lack of association encountered. Future studies should therefore aim to include FM populations with varying degrees of sleep disturbance, as well as control groups with and without insomnia, to better elucidate the role of sleep quality in fibro fog.

Interestingly, our results also reinforce the functional relevance of cognitive impairment in FM, reflected by worse quality of life scores and greater disease impact, consistent with studies showing that cognitive symptoms significantly predict work disability, social participation, and treatment adherence [8]. The identification of cognitive dysfunction as an independent predictor of greater disease impact highlights the relevance of this study and should prompt attention to the importance of its screening in this population.

We acknowledge some limitations to our study. First, the cross-sectional design prevents causal inference between pain, mood disturbances, and cognitive dysfunction. Our sample size was modest and restricted to women, which may limit the generalizability of our results, although it provides a more homogenous study population. Moreover, while the MoCA offers a rapid and accessible screening approach, it cannot substitute for detailed neuropsychological batteries in fully characterizing the cognitive profile of FM, nor does it capture subtle, domain-specific impairments. Nevertheless, the use of a validated instrument with demonstrated sensitivity, that is simple to administer and widely available in clinical practice, represents a pragmatic contribution. By supporting the feasibility of routine cognitive screening in FM, particularly in patients reporting difficulties with memory, attention, or multitasking, our findings emphasize the clinical value of MoCA as an initial step. Positive screens should then prompt comprehensive evaluation, including domain-specific testing and assessment of modifiable contributors, such as depressive symptoms and poorly controlled pain.

## 5. Conclusions

Taken together, our findings highlight the burden of cognitive dysfunction in FM, reflected by its high prevalence and its strong association with overall disease impact. These results underscore the need for systematic cognitive screening in this population. Identifying pain severity and depression as independent predictors of cognitive impairment further suggests that integrated therapeutic approaches addressing pain, mood, and cognition may improve patient outcomes. Future longitudinal studies are warranted to clarify the trajectory of cognitive dysfunction and its responsiveness to targeted interventions and should also explore the role of MoCA not only as a screening tool in clinical practice, but also as an outcome measure in interventional research.

## Figures and Tables

**Table 1:** Sociodemographic and clinical data of the participants.

Sociodemographic and Clinical Data	Fibromyalgia Group (N = 47)	Control Group (N = 19)	Univariate *p*-Value
Age (median, IQR)	49.0 [43.0–55.0]	50.0 [44.0–55.5]	0.18
**Educational level (N, %)**			
>elementary and middle school	12.00 (25.53)	8.00 (42.11)	0.24
>High School and University	35.00 (74.46)	11.00 (57.89)	
Unemployed (N, %)	15.00 (31.90)	0.00 (0.00)	<0.01
Tobacco (N, %)	11.00 (23.40)	2.00 (10.50)	0.23
Alcohol abuse (N, %)	4.00 (8.50)	0.00 (0.00)	0.32
**Physical activity (N, %)**			
>No physical activity	14.00 (29.80)	5.00 (26.30)	0.68
>Less than 150 min	10.00 (21.30)	6.00 (31.60)	
>Equal/superior to 150 min	23.00 (48.90)	8.00 (42.10)	
**Chronic medication (N, %)**			
>anxiolytic	23.00 (49.00)	0.00 (0.00)	<0.001
>antidepressants	37.00 (78.70)	2.00 (10.50)	<0.001
>muscular relaxants	25.00 (53.20)	0.00 (0.00)	<0.001
>neuromodulators (Gabapentin/pregabalin)	22.00 (46.80)	0.00 (0.00)	<0.001
>opioids	23.00 (49.00)	0.00 (0.00)	0.01
>paracetamol	13.00 (27.70)	0.00 (0.00)	0.01
>Non-steroids anti-inflammatory drugs	19.00 (40.40)	0.00 (0.00)	0.01
**Questionnaires**			
Visual Analog Scale—Pain (median, IQR)	7.0 [6.0–8.5]	0.00 [0.00]	<0.001
Visual Analog Scale—Fatigue (median, IQR)	8.0 [1.5]	0.00 [0.00]	<0.001
MoCA (median, IQR)	23.00 [5.00]	28.00 [2.00]	<0.001
MoCA < 26 points (N, %)	34.00 (72.30)	1.00 (5.30)	<0.001
HADS depression (mean, SD)	10.23 ± 4.94	3.32 ± 3.76	<0.001
HADS depression ≥ 7 (N, %)	33.00 (70.20)	4.00 (21.10)	<0.001
HADS anxiety (mean, SD)	11.72 ± 4.08	5.53 ± 3.13	<0.001
HADS anxiety ≥ 7 (N, %)	41.00 (87.20)	8.00 (42.10)	<0.001
FACIT-F (median, IQR)	19.00 [11.00]	48.00 [11.00]	<0.001
FIQ-P (mean, SD)	67.67 ± 12.12	NA	NA
EQ5D-5L total	0.64 [0.90]	1.00 [0.09]	<0.001
Pittsburgh Sleep Quality Index (mean, SD)	14.36 ± 2.82	7.26 ± 3.94	<0.001

**Legend:** FACIT-F—Functional Assessment of Chronic Illness Therapy—Fatigue; FIQ-P—Fibromyalgia Impact Questionnaire validated for the Portuguese population; HADS—Hospital Anxiety and Depression Scale; IQR—interquartile range; MoCA—Montreal Cognitive Assessment; N—number of participants; PSQI—Pittsburgh Sleep Quality Index; SD—standard deviation.

## Data Availability

The data that support the findings of this study are available from the corresponding author upon reasonable request due to ethical reasons.
